# Elemental Composition of Commercially Available Cannabis Rolling Papers

**DOI:** 10.1021/acsomega.3c09580

**Published:** 2024-04-20

**Authors:** Derek Wright, Michelle M. Jarvie, Benjamin Southwell, Carmen Kincaid, Judy Westrick, S. Sameera Perera, David Edwards, Robert B. Cody

**Affiliations:** † School of Chemistry, Environmental, and Geosciences, Lake Superior State University, 650 W. Easterday Avenue, Sault Ste. Marie, Michigan 49783, United States; ‡ Lumigen Instrument Center, Wayne State University, A. Paul Schaap Chemistry Building, 5101 Cass Avenue, Detroit, Michigan 48202, United States; § JEOL USA, 11 Dearborn Road, Peabody, Massachusetts 01960, United States

## Abstract

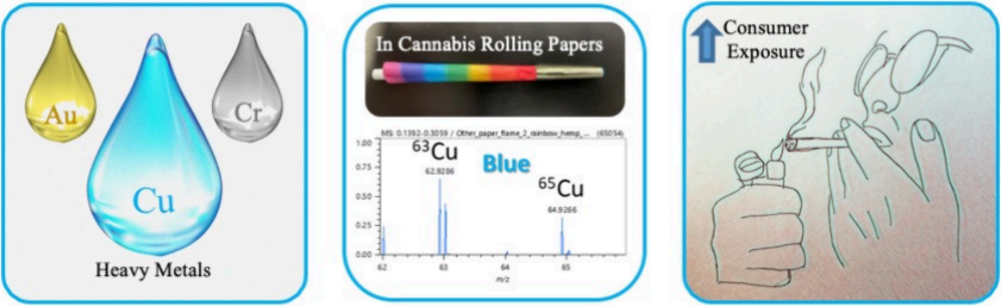

With the recent legalization of cannabis in multiple jurisdictions and widespread use as a medical treatment, there has been an increased focus on product safety and the potential impacts of contaminants on human health. One factor that has received little attention is the possible exposure to potentially hazardous levels of toxic elements from rolling (smoking) papers. The elemental composition of rolling papers is largely unregulated, with a minority of jurisdictions regulating papers only when they are part of a final cannabis product. This study reports the concentrations of 26 elements in commercially available rolling papers and estimates potential maximum exposures relative to USP232 and ICH Q3D dosages in pharmaceutical compounds. Exposure estimates indicate that the concentrations of several elements in some products, particularly Cu, Cr, and V, may present a potential hazard to frequent users. Several elements, including Ag, Ca, Ba, Cu, Ti, Cr, Sb, and possibly others, are likely present in elevated quantities in some papers due to product design and manufacturing processes. Our results further suggest that Cu-based pigments are used by a number of manufacturers and that regular use of these products might result in exposures as high as 4.5–11 times the maximum exposure limits. Further research to quantify the contribution of rolling papers to elemental exposure under realistic smoking conditions is warranted.

## Introduction

Reported cannabis use in the United States (U.S.) has been on the rise, with 49% of American adults saying they have tried cannabis in some form, up from 34% in 2012.^1^ Among cannabis users, 12% say they consume mostly through “smoking”, which has held steady at 11–13% in recent years but has increased from 7% in 2013 when they were first surveyed.^1^ In the United States, cannabis carries two federally defined definitions. Cannabis containing less than 0.3% tetrahydrocannabinol (THC) is classified as industrial hemp, while cannabis containing more than 0.3% THC is classified as marijuana. Currently, the majority of regulatory standards apply to marijuana. As hemp is biologically the same plant as marijuana (but with <0.3% THC), both will be designated as cannabis hereafter. While marijuana use remains federally illegal in the U.S., as of 2023, all but 12 U.S. states have medical and/or recreational cannabis use legalization laws. As of mid-2021, about 2.3% of the U.S. population were registered medical cannabis patients.^2^ Medical use patients are those that have a qualifying health issue and have been prescribed cannabis to treat the symptoms of their condition.^3^ Qualifying health issues vary among jurisdictions but typically include terminal illness, HIV/AIDS, autism, cancer, Crohn’s disease, glaucoma, seizure disorders, persistent nausea, and other debilitating diseases and symptoms.^3^

The disparity between the state and federal legality of cannabis in the United States has led to individual states determining the regulatory limits for tested products available in legal dispensaries. To date, there have been few studies to determine a universal standard for limits of action in cannabis products, but many states have borrowed from guidelines established by the U.S. Pharmacopeial Convention (USP).^4^ The USP is a nonprofit organization that creates quality assurance standards for medicines, dietary supplements, and food, which serve as useful guidelines for acceptable exposure limits and often as a basis for legal exposure limits.^5^ Thus, compliance analysis is important to protect the health and safety of consumers, especially users who may have a weakened immune system.^4,6^

Cannabis intended for commercial sale generally undergoes full compliance testing, the parameters of which vary between the products. Analytical testing includes pesticide residues, residual solvents, heavy metals, and microbes and may include foreign matter, terpenes, and mycotoxins. The regulatory limits for all tested categories vary from state to state and sometimes between medical- and recreational-grade cannabis products within a state. For example, in the state of Michigan, the action limits are the same for medical and recreational grade cannabis in all categories except for total yeast and mold count on bud, shake/trim, and kief, where the action limit is 10,000 CFU g^–1^ (colony forming units) for medical-use cannabis and 100,000 CFU g^–1^ for recreational-use cannabis.^7^ In comparison, action limits are 100 CFU g^–1^ in California and 10,000 CFU g^–1^ in Colorado.^8^

Like microbial action limits, heavy metal action limits vary greatly from state to state as well as which heavy metals are included in testing. For example, the states of California, Arizona, and Colorado have tested for four heavy metals: arsenic, cadmium, mercury, and lead. Washington DC. tested for those four as well as chromium, silver, and barium. The states of Michigan and New York add nickel and copper testing to those previously mentioned, but Michigan tests for copper only in inhaled concentrates, not in flowers, regardless of its end use. The regulatory limits for lead, for example, range from 0.5 μg g^–1^ in the states of California and New York to <10 μg g^–1^ in Colorado.

Heavy metal exposure through inhalation poses a long-term health risk of accumulation in the body.^8,9^ Heavy metals are toxic and carcinogenic and can cause a variety of diseases.^9^ For example, chronic exposure to cadmium can result in kidney, bone, and lung disease,^10^ and McGraw et al.^11^ found significantly higher levels of cadmium in urine and blood from marijuana smokers and cigarette smokers compared to all nonsmokers. Elevated levels of copper, lead, and zinc in the body can lead to neurodegenerative diseases.^12^ Consuming cannabis through combustion (smoking) poses the greatest risk to human health as studies have shown cannabis smoke to contain all of the mentioned heavy metals as well as selenium.^13,14^

There is a common perception that if a batch of cannabis flower has passed heavy metal analysis, subsequent products made from that flower would also pass, but this may not be the case for prerolls. Prerolls are ready-to-smoke joints that consist of cannabis flower, rolling paper, and a filter (a piece of folded paper to prevent Cannabis flower from entering the mouth during smoking).^15^ Prerolls are a popular and relatively inexpensive way to buy and consume cannabis from dispensaries. Prerolls most commonly come with one gram (g) of cannabis flower but can range from 0.5 to 3 g.^15^ In 2020, a cannabis testing lab in the state of California determined that finished prerolls made from cannabis flower that had previously passed heavy metal and pesticide testing were failing above the action limits.^16^ Further investigation found that heavy metals in the rolling papers caused the failures. Out of 101 papers, cones, and wraps tested, 91 had detectable levels of at least one heavy metal (cadmium, lead, arsenic, or mercury), and 8 had detections over California action limits.^16^ While this report was circulated widely in the cannabis industry, we are not aware of any studies published in the peer-reviewed literature.

Similar research on heavy metals in tobacco cigarettes led to the discovery that tipping papers, the part of the cigarette that touches a smoker’s lips, and filters contribute trace heavy metals^17^ and that different types of rolling papers (slow, medium, or fast-burning, bleached, flavored, and wood cellulose vs other plant cellulose) contain different, and not insignificant, amounts of toxic elements.^18^ Several studies also revealed that the heavy metal content in cigarette rolling paper varies significantly (Table 1).

**Table 1:** Previous Studies That Examined the Heavy Metal Content of Cigarette Rolling Papers, Tipping Paper,^a^ and Cannabis Rolling Papers or Cones^b^^16−22^

study	element	heavy metal content in paper
Wu et al., 1997	As	0.01 μg/cigarette
	Cd	0.04 μg/cigarette
	Zn	0.4 μg/cigarette
Suo et al., 2008	As	0.159 μg g^–1^
	Cd	0.107 μg g^–1^
	Cr	1.908 μg g^–1^
	Cu	2.466 μg g^–1^
	Ni	1.573 μg g^–1^
	Pb	0.411 μg g^–1^
Li et al., 2016	As	0.036–0.126 μg/cigarette
	Cd	0.0001–0.02 μg/cigarette
	Cr	0.105–0.2 μg/cigarette
	Hg	0 μg/cigarette
	Ni	0.09–0.14 μg/cigarette
	Pb	0.04–0.79 μg/cigarette
Zumbado et al., 2019	Ag	0.005–0.05 μg g^–1^
	As	0.07–0.144 μg g^–1^
	Cd	0.003–0.005 μg g^–1^
	Cr	0.79–1.76 μg g^–1^
	Hg	0.029–0.037 μg g^–1^
	Ni	0.62–1.62 μg g^–1^
	Pb	0.17–0.27 μg g^–1^
Cheng et al., 2021^a^	As	0.05–0.3 μg g^–1^
	Cr	0.44–10.2 μg g^–1^
	Hg	0.0003–0.003 μg g^–1^
	Ni	0.23–0.84 μg g^–1^
	Pb	0.20–0.56 μg g^–1^
Dihn et al., 2021	Cd	0.08 ± 0.11 μg g^–1^
	Hg	<LOD
	Pb	0.25 ± 0.24 μg g^–1^
SC Laboratories, 2020^b^	As	1.6–3.2 μg g^–1^
	Cd	0.56 μg g^–1^
	Pb	0.9–60.3 μg g^–1^

a Tipping paper only.

b Cannabis rolling papers/cones.

Heavy metals in cigarette rolling papers can be attributed to residual chemicals, additives, and contaminants from the manufacturing process,^17^ ink and pigments used in tipping paper,^17^ and the use of pulp from plants cultivated in contaminated soil.^18^ Using recycled paper may pose an even greater risk as the recycling process requires additional additives to improve paper surface and color.^17,23^ These additives may include, in particular, lead, arsenic, cadmium, chromium, and zinc.^24^

In states like Michigan, final form testing is not required for cannabis prerolls if the flower used in the preroll has passed full compliance testing before creating the preroll.^25^ This creates a situation in which heavy metal contributions from rolling paper materials may escape the compliance analysis process. This study aimed to quantify the heavy metal content of commercially available cannabis rolling paper materials. Specifically, we sought to (1) characterize the elemental composition of a selection of commercially available rolling papers that may be used by cannabis consumers, (2) evaluate the potential for exposure risk in relation to accepted standards, and (3) identify strategies that can be implemented by manufacturers and regulators to minimize potential consumer exposure.

## Results and Discussion

### Elemental Composition of Rolling Papers

**Figure 1:**
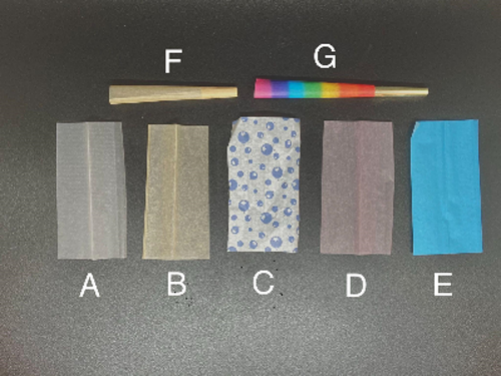
Examples of types of rolling papers and cones tested: (A) unspecified material, bleached; (B) hemp, unbleached; (C) hemp, blue print; (D) wood pulp, pink; (E) hemp, blue; (F) bamboo, unbleached; (G) palm pulp, rainbow with a metallic tip.

For the Cannabis rolling papers examined in this study (representative examples shown in Figure 1), the mean, median, and ranges of each of the 26 elements quantified by ICP-MS were calculated (Table 2) and compared to regulatory limits for legal cannabis products for consumption by inhalation from various US states and Canada for comparison. While the elemental composition of the papers themselves is typically not subject to these regulatory limits, they serve as a useful point of reference for evaluating the potential contribution of rolling papers to consumer exposure. Additionally, in jurisdictions such as California that regulate prerolls as a final product, rolling papers may become subject to regulatory compliance as a component of the final product.

**Table 2:** Elemental Composition of Rolling Papers in Comparison with Regulatory Limits for Elements in Cannabis^a^

units	element	MM	median	mean	max	CA	MI	NY	CO	AZ	Wash. DC.	Canada
mg g^–1^	Al	<0.01	0.09	0.38	2.9							
	Ca	0.05	26	31	116							
	Fe	<0.01	0.07	0.10	0.72							
	K	0.01	0.20	1.0	16							
	Mg	0.01	0.52	1.3	14							
	Na	0.02	0.31	0.78	6.5							
μg^–1^	Ag	<0.01	<0.01	3.1	161						1.4	
	As	<0.01	0.04	0.07	0.24	1.5	0.4	0.2	<10.0	0.4	0.4	0.2
	8a	0.22	3.9	13	149						60.0	
	Be	<0.01	<0.01	<0.01	0.04							
	Cd	<0.01	0.02	0.03	0.14	0.5	0.4	0.3	<4.1	0.4	0.4	0.2
	Co	<0.01	0.03	0.12	3.1							
	Cu	<0.1	2.9	31	251		3′	30.0				
	Cr	<0.1	1.2	1.7	8.5		1.2	0.3			0.6	
	Hg	<0.002	<0.002	0.013	0.17	0.1	0.2	0.1	<2.0	1.2	0.2	0.2
	Mn	0.09	14	17	138							
	Mo	<0.1	<0.1	0.95	33							
	NI	<0.1	0.35	0.53	4.9		1.0	0.5				
	Pb	0.01	0.17	0.22	1.2	0.5	1.0	0.5	<10.0	1.0	<1.0	0.5
	Sb	<0.01	<0.01	1.4	11.72			2.0				
	Se	<0.2	<0.2	<0.2	<0.2							
	Th	<0.01	0.02	0.03	0.17							
	Ti	<0.2	<0.2	0.11	0.45							
	U	<0.01	0.04	0.06	0.21							
	V	<0.01	0.19	0.43	5.3							
	Zn	<0.1	1.8	6.0	42							

a Michigan does not regulate Cu in cannabis plant material, only in vape liquids.

Within the context of the observed concentrations of metals in rolling papers, the degree of variability in how various jurisdictions regulate acceptable concentrations of toxic elements, such as As, Cd, Hg, and Pb, is particularly notable. Even if rolling papers were regulated similarly to cannabis, the regulatory limits for these elements vary by a factor of 20–50 fold between jurisdictions, with no clear pattern. Further, the list of regulated elements beyond these four, if any, as well as their acceptable concentrations, varies even more considerably. Based on these observations, it is not clear that all jurisdictions are employing sound risk assessment principles in these standards. In any case, the lack of consensus on acceptable limits for various elements in the cannabis product should be carefully considered when interpreting these results.

**Figure 2:**
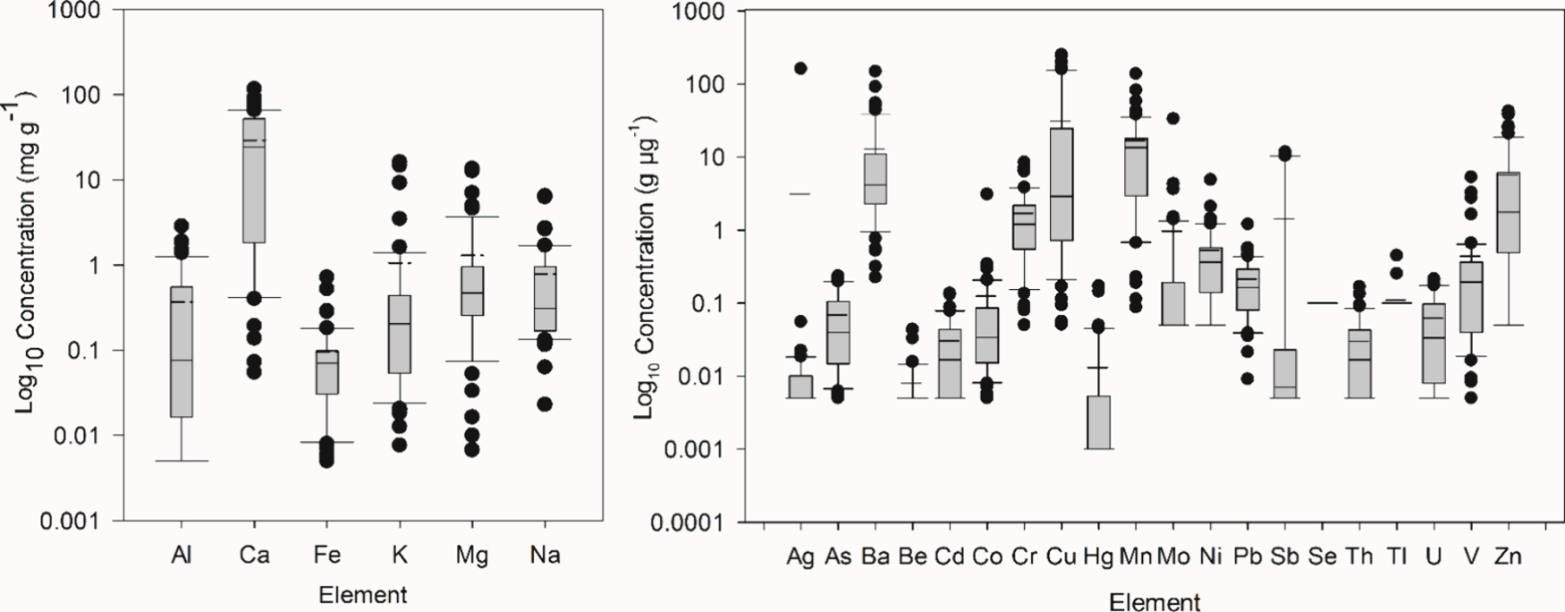
Distribution of elements in rolling paper samples where the box represents the interquartile range; the whiskers indicate the minimum and maximum values; the solid line inside each box represents the median; the dashed line indicates the mean; and the dots represent outliers.

In the set of samples studied, the major inorganic elemental constituents of rolling papers were typically Ca ≫ Mg > Na > K > Al > Fe ≫ Mn (Figure 2). Elemental composition was typically consistent with a log-normal distribution, with the exception of Ca and Mn, which were neither normally distributed (*p* = 0.02 and *p* < 0.01) or log-normally distributed (*p* = 0.03 and *p* = 0.03). In the case of Ca, this may be due to the use of Ca-based additives such as CaCO_3_, which is frequently used as an inorganic filler in paper manufacturing.^26−28^ Variability in Mn is more likely due to variations in the source fibers, as Mn uptake and tissue concentration can vary substantially by species, environmental/growth conditions, etc.^29−31^ Ba, Cu, and Zn were also relatively abundant. Ba compounds might be used as a whitening additive or opacifying agent similar to CaCO_3_ by some manufacturers.^32^ Conversely, the Cu content appeared to be correlated primarily with the use of colored inks. The potential source of Zn was unclear, though it was noted that several of the flavored samples were also some of the higher concentrations observed. This suggests the possibility that manufacturing practices could also potentially contribute to Zn concentrations, but the evidence is inconclusive.

### Arsenic, Cadmium, Mercury, and Lead

Arsenic, cadmium, mercury, and lead are commonly considered to be the elements of greatest human exposure concern. As a result, these elements are always required to be monitored in commercial pharmaceutical compounds in the U.S.^33^ and the European Union.^34^ and all of the jurisdictions that regulate heavy metals in cannabis products have established limits for these elements. In the samples studied, there was significant variation in the concentration of all four elements with measured concentrations spanning 2–3 orders of magnitude. The greatest variation was seen with Pb, with the highest sample having a concentration of 1.2 μg g^–1^ and two others exceeding 0.5 μg g^–1^. While this is significantly lower than the maximum value of 60.3 μg g^–1^ reported by SC Laboratories,^16^ it is sufficiently elevated that it could potentially lead to product failure if final product testing of prerolled products is required (California action level for Pb of 0.5 μg g^–1^).

While none of the samples exceeded the California limit for arsenic, four of the samples contained As at 0.2 μg g^–1^ or higher, with three more between 0.15 and 0.2 μg g^–1^. As most of the other jurisdictions surveyed have adopted limits for As of 0.2–0.4 μg g^–1^, this suggests that if California were to adopt more restrictive regulatory limits, the As content of rolling papers might lead to increased product failures. Similarly, while none of the samples measured in this study exceeded regulatory limits for cadmium in any of the jurisdictions, three samples contained Cd ∼0.13 to 0.14 μg g^–1^, just slightly lower than the Canadian limit of 0.2 μg g^–1^. Additionally, two samples exceeded California’s action level for Hg (0.1 μg g^–1^), but both were <0.2 μg g^–1^.

### Exposure Potential

In order to assess the potential human health risks to cannabis consumers from potentially toxic elements in rolling papers, we calculated the exposure potentials for both a 2 and 5 g d^–1^ smoker (Table 3) and compared the values to reference values for inhaled pharmaceuticals from USP 232 (As, Cd, Cu, Cr, Hg, Mo, Ni, and Pb) or ICH Q3D (Ag, Ba, Co, Sb, Tl, and V). The results of this analysis suggest that several elements that are typically unregulated in consumer cannabis have the potential to contribute to significant exposures when smoked from certain rolling papers. Further, there is evidence that in common use, many consumers may fill papers to around half capacity, which would increase the mass of paper smoked per gram of cannabis consumed.^35^ With the exception of Cd and Tl, all of the elements considered here had the potential to make a meaningful contribution to consumer exposure from at least one type of rolling paper if used consistently by a very heavy smoker (5 g d^–1^), considering that cannabis itself might be expected to contribute significant additional exposure. Even without the additional exposure from added cannabis, it is clear that there is substantial exposure potential from Cu, Cr, and V in rolling papers, while several other elements (Ag, Ni, Co, Mo, Sb, etc.) might be problematic in certain instances. The sources of many of these elements in rolling papers are unclear, but they could be due to a variety of sources, such as uptake from contaminated soil, air, or water pollution. Alternatively, several of these are commonly found as alloying elements in various grades of steel or stainless steel (Ni, Cr, V, Mo, Co), which might be utilized in harvesting or production facilities.

**Table 3:**
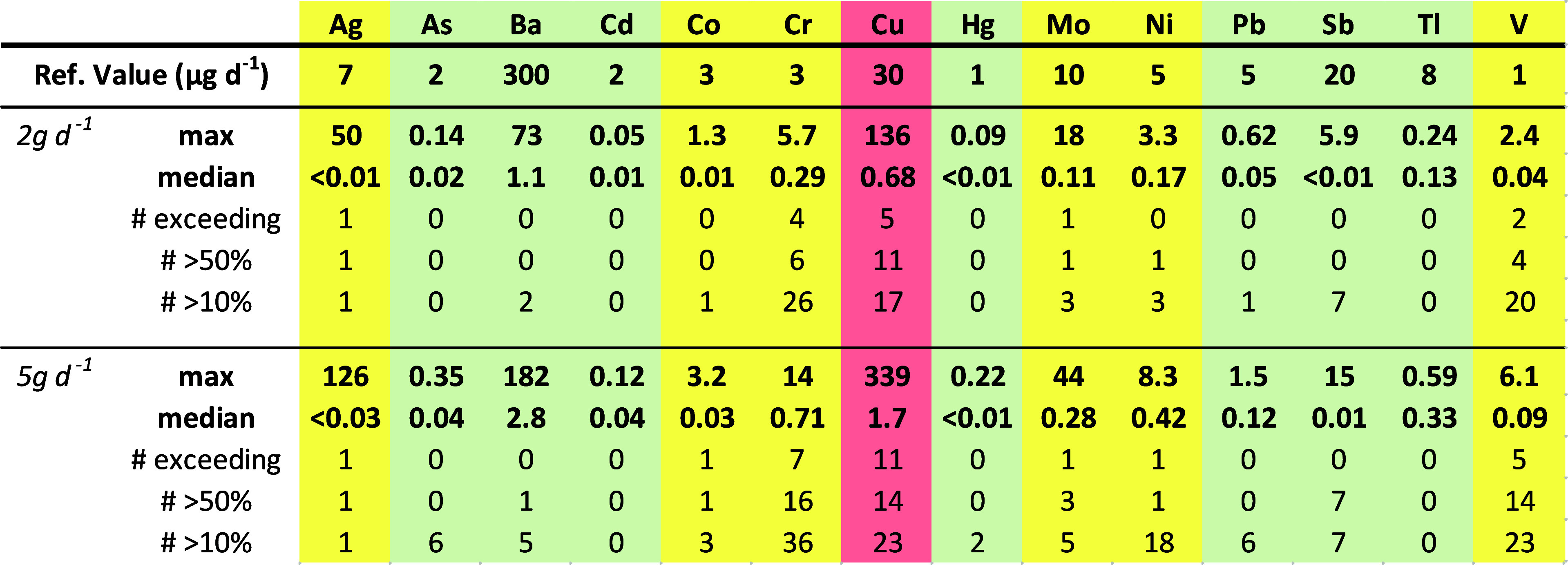
Exposure Potentials of Rolling Paper Samples Relative to Reference Exposure Values from USP 232 or ICH Q3D^a^

a Exposure potentials (μg d^–1^) are calculated for both a 2 and a 5 g d^–1^ smoker and a number of samples exceeding the reference value, >50% of the reference value, and >10% of the reference value are shown (*n* = 53). Red shading indicates elements where the exposure potentials of multiple products exceed the reference dose and the maximum observed concentration exceeds the reference dose at 2 g d^–1^ consumption by a factor of five or more. Yellow shading indicates that at least one product exceeds the reference dose, and green indicates that no products exceeded the reference dose.

Copper, in particular, was found at elevated concentrations, >30 μg g^–1^, in ∼1/4 of the samples tested. In the case of the sample with the highest Cu concentration (250 μg g^–1^), a single paper contained 71 μg of copper, yielding more than twice the USP limit without the added cannabis. This sample is only one of four tested that exceed 30 μg in a single paper; three paper cones and the 24k gold cone. Further exposure from the cannabis itself might potentially increase the daily exposure significantly. Unfortunately, most current data on Cu in cannabis comes from phytoremediation studies on contaminated soil, but unpublished data from an unrelated study on field-cultivated cannabis (industrial hemp for CBD production) found typical concentrations of 10–20 μg g^–1^ Cu (median = 14 μg g^–1^, *n* = 73; Wright et al., unpublished data). If these values are approximately representative of consumer cannabis, a 2 g d^–1^ smoker would be exposed to an additional 20–40 μg d^–1^, and a 5 g d^–1^ smoker would be exposed to an additional 50–100 μg d^–1^. Inhalation of Cu has been shown to cause pulmonary inflammation, altered gene expression, and cytotoxicity^36,37^ Copper also plays a significant role in the onset of neurodegenerative diseases like Alzheimer’s and Wilson’s disease.^12^ The levels of Cu in many of these samples are a potential cause for concern, and their implications for public health should be further investigated.

It is also worth noting that the 24k gold wrap contained a significant quantity of Ag (38 μg), more than five times the acceptable daily dosage under ICH Q3D. Additionally, this sample presumably contains a substantial mass of gold (not quantified), as gold likely contributes a significant fraction of the 0.23 g total sample mass. If the manufacturer’s purity claim of 24k gold (99.95% purity) is valid, this sample should contain a minimum of 138 mg Au. It is quite possible that this particular sample is relatively inert under normal smoking conditions. However, as inhalation of Au nanoparticles has been shown to result in translocation to secondary organs and bioactivity with size-dependent effects and cytotoxicity,^38−40^ an a priori assumption of low risk seems premature.

### Sources of Metals: Impact of Product Design and Manufacturing Practices

Due to the highly elevated metal concentrations and resulting exposure risk we observed in some of the samples, we decided to further characterize a subset of the samples using additional methods. Based on visual examination and further examination under both a stereomicroscope and reflected light microscope, we suspected that several manufacturers were utilizing inks containing Cu pigments. Our objective was to determine if the source of elevated metal concentrations was related to product design (raw materials or additives) or resulted from inadvertent contamination during production and manufacturing. In particular, we focused on Cu for two reasons: (1) elevated copper concentrations occurred in a significant number of samples, and (2) we observed that with the expectation of the 24k gold cone, all of the samples that contained copper >30 μg g^–1^ were colored with blue pigment or another color which might include blue pigment as an ingredient (green, purple, and black).

**Figure 3:**
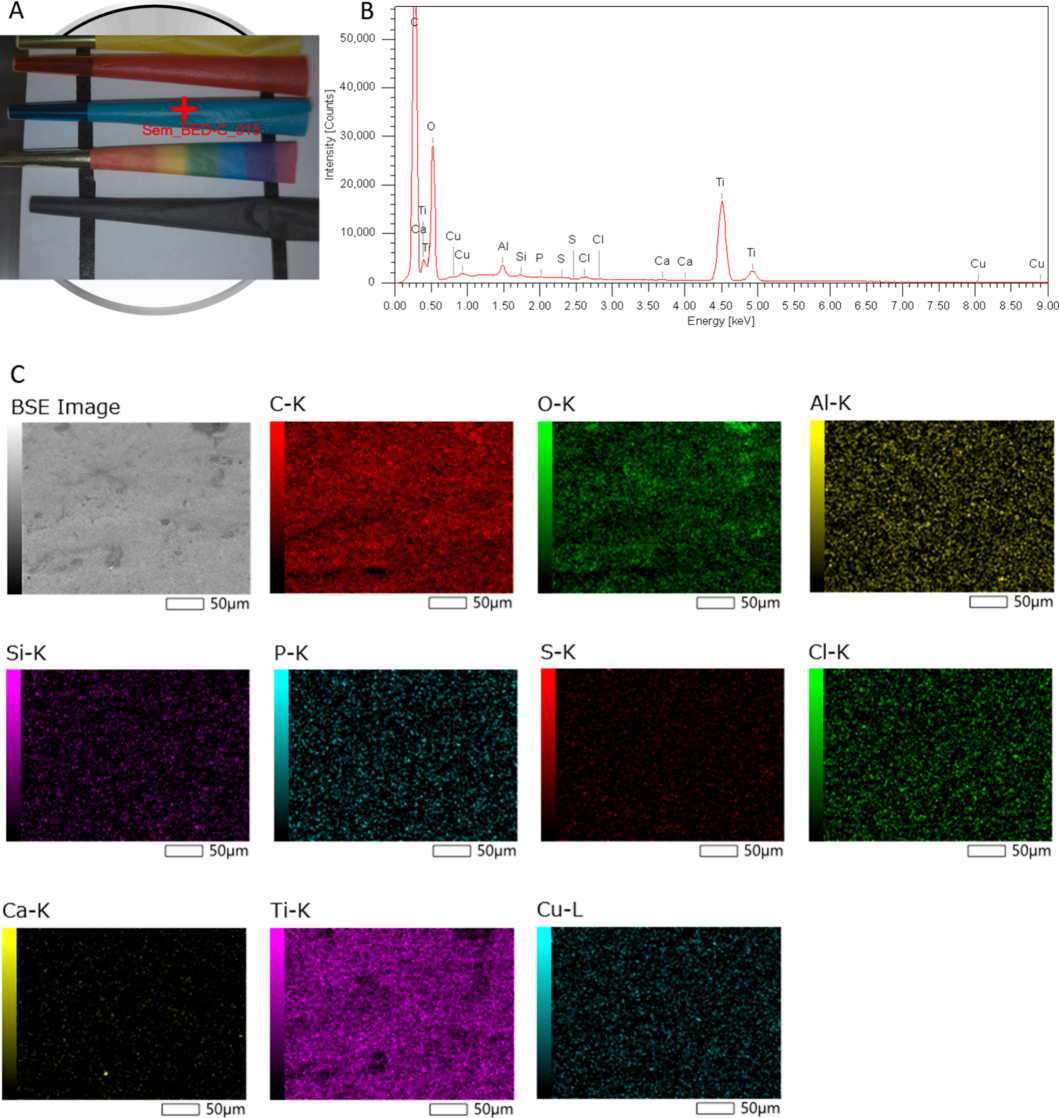
SEM-EDS analysis of the blue cone. (A) Photograph of sample analysis location. (B) EDS spectrum showing elements detected. (C) BSE image and EDS maps showing element distributions.

To further investigate this hypothesis, we examined five colored cones from two different manufacturers (Manufacturer 1: yellow, red, blue, and black; Manufacturer 2: rainbow/multicolored stripes) by SEM–EDS coupled with backscattered electron imaging (atomic number contrast) (Figure 3). EDS mapping of the yellow cone (Cu = 0.77 μg g^–1^) detected no Cu as expected. However, a significant amount of titanium was detected dispersed evenly across the surface, likely indicating the use of TiO_2_ as a whitening/opacifying agent. EDS analysis of the red cone (Cu = 0.74 μg g^–1^) yielded similar results, again identifying TiO_2_ with the addition of surface particles containing Sr and S. This suggests the use of the strontium salt of one of the red organic dyes as the coloring agent. EDS analysis of the blue cone (Cu = 251 μg g^–1^) showed Ti and Cu evenly distributed across the surface. The limited sensitivity of EDS resulted in the copper peak being quite small and barely above the background. This is due to the dispersal of Cu across the surface in a thin layer. Had Cu-enriched particles been responsible for the elevated Cu in the sample, they would have been large enough and abundant enough to be easily detectable. As no distinct Cu-containing particles were visible by either backscatter imaging or EDS mapping, this supports the use of a Cu-containing pigment, as opposed to manufacturing contamination. Additionally, the large difference in Cu content between cones from the same manufacturer based on pigment color (cones that were blue or green, or colors such as purple, which may also contain blue ink), contained the highest Cu concentrations, suggesting that the underlying paper material was not the source of highly elevated Cu in these samples. SEM analysis of the black cone (Cu = 43 μg g^–1^) did not detect dispersed Cu as it was below the expected detection limit, but neither did it detect any copper-containing particulate matter.

**Figure 4:**
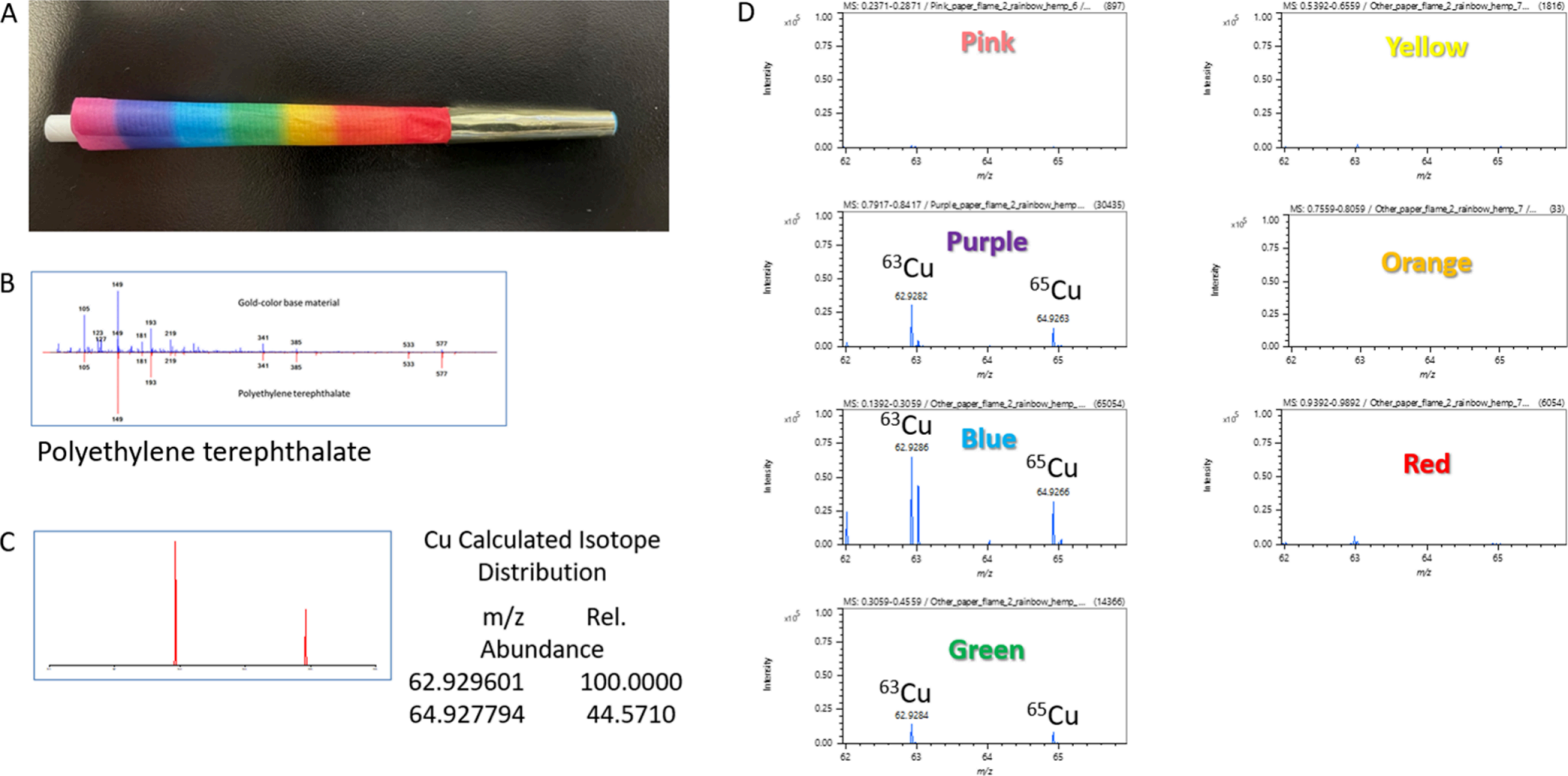
Thermal desorption/pyrolysis DART-TOF analysis of the rainbow cone. (A) Photograph of the sample. (B) Mass spectrum from analysis of the gold-colored tip. (C) Copper isotope data showing isotopic fidelity. (D) Mass spectra from different color regions.

Finally, the rainbow cone (Cu = 243 μg g^–1^) was analyzed across its length from tip to end. The tip was metallic gold-colored with no detectable Cu, though it did contain detectable Cr. This particular sample had the fourth highest Cr content (6.3 μg g^–1^). Its apparent localization in the metallic-colored tip suggested that Cr might be a component of the metal layer, although EDS peak intensities were low. Cu peaks were not detected by EDS in the red, orange, yellow, and pink portions of the cone, but small Cu peaks were detected in the green, blue, and purple portions. Due to the limited detection capabilities of SEM-EDS, we subjected the rainbow cone to a more sensitive analysis using thermal desorption/pyrolysis DART-TOF-MS (Figure 4). The findings of the SEM-EDS analysis were confirmed, with Cu detected with isotopic fidelity in only the green, blue, and purple portions, with the highest signal originating from the blue region. Additionally, we analyzed the metallic-colored tip materials in the rainbow (gold-colored tip) and blue (silver-colored tip) cones and identified that the tips were composed of poly(ethylene terephthalate) (PETE) with significant quantities of antimony. Antimony is used as a catalyst in PETE production and is therefore found as a contaminant in PETE and may also be added in additional quantities as a flame retardant synergist.^41^ Combustion fumes of PETE have been shown to contain a variety of potentially hazardous compounds such as formaldehyde, methanol, acetone, and benzene,^42^ and thus, may constitute an additional hazard in addition to their elemental constituents if combusted. Additionally, DART-TOF analysis confirmed the presence of Cr in the gold-colored tip of the rainbow paper, but no Cr was detected in the silver-colored tips of the blue cone.

**Figure 5:**
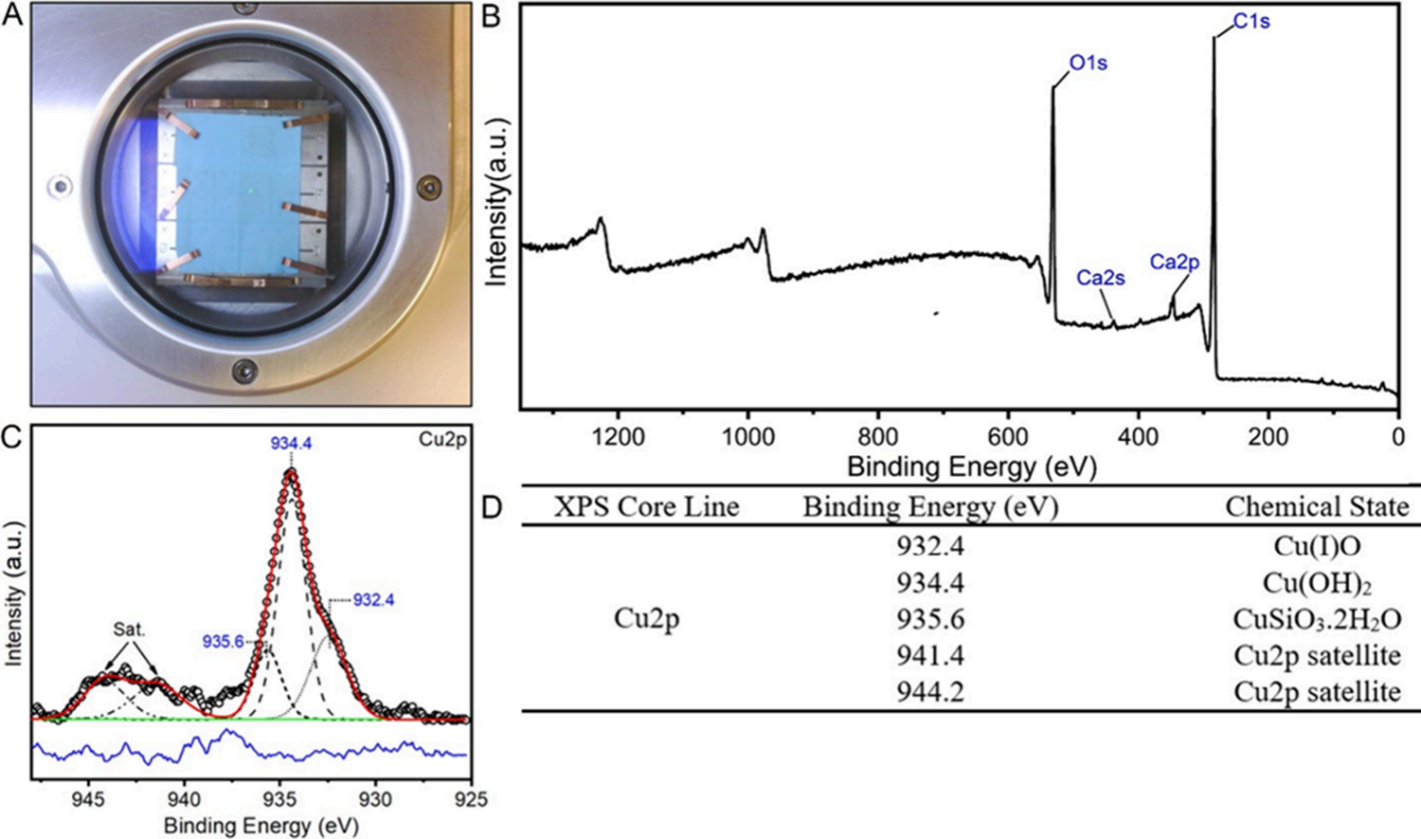
XPS analysis of the blue paper sample. (A) Photograph of the sample. (B) XPS survey spectrum. (C) Peak fit to the high-resolution Cu 2p spectrum. (D) Chemical state identification of Cu.

Finally, we sought to confirm the chemical identity of the blue pigment to the degree possible, so we analyzed one of the blue papers (Cu = 160 μg g^–1^) using X-ray photoelectron spectroscopy (Figure 5). Numerous blue colored copper-containing pigments have been used historically and/or are currently available in commerce including both inorganic pigments (i.e., basic copper carbonate: Cu_2_(OH)_2_CO_3_, Egyptian blue: CaCuSi_4_O_10_, Azurite: (Cu_3_(CO_3_)_2_(OH)_2_), Han blue: BaCuSi_4_O_10_, and organometallic pigments (i.e., copper phthalocyanine). XPS analysis confirmed the distribution of Cu across the surface of the sample and identified CuO, Cu(OH)_2_¸, and CuSiO_3_·2H_2_O, with Cu(OH)_2_ as the dominant species. This is visually consistent with the observed coloration of the sample and is further evidence for the use of Cu-based pigments.

## Conclusions

We have presented the first data on the elemental composition of rolling papers used for cannabis consumption that consider a broad suite of elements. There is significant variability in the concentrations found as well as potential exposure to consumers when utilizing a maximum exposure model. Under the current regulatory scheme, rolling papers are virtually unregulated, except in a limited number of jurisdictions, such as the state of California, as part of a prerolled final product. This general lack of regulation is of concern in light of their potential to substantially increase exposure to several potentially toxic elements, particularly copper. This is of even greater concern considering the widespread medical use of cannabis by at-risk populations.

Due to the disparity between the state and federal legality of cannabis in the United States, there is currently no guidance from federal agencies such as the US Food and Drug Administration, leading to a fragmented regulatory approach. Additional efforts by state regulatory agencies to reach a consensus on limits to toxic elements in cannabis and smoking papers are warranted based on our findings, as is additional research to determine exposures based on realistic use patterns.

Our findings also show that product design and manufacturing practices have the potential to significantly increase exposure and have documented that the use of copper-based printing inks appears to be common. Elimination of the use of Cu-containing inks by manufacturers would reduce the median Cu concentration in our data set from 30 to 3 μg g^–1^ and would eliminate all cases where smoking the papers alone may potentially exceed USP and ICH Q3D daily exposure limits. Additionally, we documented the use of Ag-, Sb-, and Cr-containing PETE in products intended to be smoked. Though the health risks of these under actual use conditions, if any, are currently unknown, manufacturers might consider the replacement of the PETE tips with clean paper as a sound precautionary measure.

## Materials and Methods

### Sample Selection

Commercially available rolling papers and cones were purchased from four retailers in Michigan between February and June 2022. Samples were selected to encompass a wide variety of products while including “popular” brands, as recommended by store managers. Products selected are widely available for purchase regionally and, in most cases, nationally or internationally, as confirmed through a brief survey of major online retailers. Product information from the label and manufacturers’ Web sites indicate fiber materials include hemp, cellulose, flax, rice, cotton mallow, bamboo, palm, goji berry, and mixtures/unspecified. Samples included major types of rolling papers (standard papers, wraps, and cones) and various product sizes, thicknesses, and flavors. One sample contained a blend of hemp and “food grade” 24k gold. Several papers/cones also appeared brightly colored (red, pink, blue, green, teal, yellow, purple, etc.), and some cones contained tips of a metallic appearance.

### ICP-MS Analysis

The concentrations of 26 elements (Ag, Al, As, Ba, Be, Ca, Cd, Co, Cr, Cu, Fe, Hg, K, Mg, Mn, Mo, Na, Ni, Pb, Sb, Se, Th, Tl, U, V, and Zn) were determined in a subsample of each paper by inductively coupled plasma mass spectrometry as follows. Samples were carefully removed from their packaging, weighed, and ∼0.2 to 0.5 g of each sample was transferred to an acid-cleaned perfluoroalkoxy alkanes (PFA) digestion vial. For rolling papers that came in paper dispenser packages, the top paper was discarded prior to sampling. For smaller and thinner papers, obtaining a sufficient mass for analysis required compositing 5–10 papers, while cones and wraps were analyzed individually. Several of the cones were constructed of visibly different materials on the tip. While we considered separating the tips prior to digestion, the entire unit was digested whole for several reasons: (1) while the tips may not be intended to be smoked, there is a possibility they may partially burn as some users may attempt to maximize cannabis consumption, (2) it was unclear whether hot air/combustion residues might liberate some portion of the material, and (3) increased sample handling might increase the likelihood of inadvertent contamination leading to biased results.

To each digestion vial containing the sample, 9 mL of concentrated nitric acid (HNO_3_) and 1 mL of concentrated hydrochloric acid (HCl) (trace metal grade acids (Aristar Plus, VWR Chemicals BDH)) were added. The vessels were subsequently capped and microwave-digested according to the manufacturer’s recommended protocol for plant material (Mars 6, CEM Corporation; Matthews, NC). Following digestion, samples were transferred to acid-cleaned 50 mL polypropylene centrifuge tubes and diluted to a final volume of 50 mL with ultrapure water. Digestion blanks were prepared identically to sample digests, except no sample was added, with at least four digestion blanks included randomly in each digestion batch. Matrix spikes were added to duplicate samples prior to digestion to assess method performance. To ensure the reproducibility of the analysis, duplicate samples were digested in each digestion batch.

Sample digests were then analyzed with an Agilent 7800 inductively coupled plasma mass spectrometer (ICP-MS) within 24 h using EPA 6020 E (modified). The instrument was configured with a concentric nebulizer (Micromist U series; Glass Expansion Inc. Pocasset, MA), a chilled spray chamber, Ni cones with a Ni-plated copper base, 4× aerosol dilution, a forward power of 1600 W, and an octopole collision cell to reduce polyatomic interferences. Tuning was performed with the manufacturer’s recommended autotune procedure using standard tuning solutions. Internal standard elements (^6^Li, Sc, Ge, Y, In, Tb, and Bi) were added online via a mixing T. Calibration was performed using a custom multielement standard (Inorganic Ventures, Christiansburg, Virginia) with verification by a second standard acquired from a separate lot. Calibration ranges were 0.001–100 mg L^–1^ for Na, K, Ca, Mg, Al, and Fe and 0.1–500 μg L^–1^ for all other elements except Hg, which was 0.002–2 μg L^–1^. On occasion, unexpectedly high concentrations of certain elements were encountered, which exceeded the normal calibration range. To confirm that these elements were within the linear range of our instrument, additional standards were analyzed as needed. Element quantification was performed using the manufacturer’s recommended isotopes, with confirmation from a second isotope when possible. Interferences from doubly charged interferences on ^66^Zn, ^75^As, and ^78^Se were monitored using the narrow peaks correction method. Minimum reportable values were assigned by calculating the quantification limit as 10 times the standard deviation of the digestion blanks, then rounding up to the nearest whole digit, and are reported in Table S1.

### SEM–EDS Analysis

Analysis of element distributions in five of the brightly pigmented cones was performed by scanning electron microscopy (JEOL JSM-IT510LA) with energy-dispersive X-ray spectroscopy (SEM-EDS). Samples were mounted on double-sided carbon tape, uncoated. Images were generated under low-vacuum conditions at 15KV using a backscattered electron detector to provide compositional (atomic number) contrast and energy-dispersive X-ray spectroscopy (EDS) to provide data on elemental composition and spatial distribution.

### DART-MS Analysis

Further analysis of the five brightly colored cones was performed by direct analysis in real time/time of flight mass spectrometry (DART-TOF) and atmospheric pressure flame ionization. The mass spectrometer (AccuTof-DART; JEOL USA, Peabody MA) was equipped with a DART-JS ion source (IonSense; Billerica, MA) and a thermal desorption/pyrolysis attachment (ionRocket; BioChromato Inc., Fujisawa, Kanagawa-ken, Japan) for the DART ion source. To identify the polymer composition of the metallic tip, a small (0.5 mm^2^) segment of the tip was placed on a copper sample holder and heated to 600 °C at a rate of 100 °C min^–1^. The pyrolysis DART mass spectra identified the polymer as polyethylene terephthalate. Qualitative elemental analysis was performed by holding small (approx. 1 mm^2^) segments of the rolling paper and metallic tip directly in front of the mass spectrometer atmospheric pressure sampling orifice and igniting the segments with a butane torch. In-source collision-induced dissociation produced positive ions for elemental copper, chromium, antimony, and simple oxides. The mass spectrometer atmospheric pressure interface potentials were: orifice 1 = 150 V, ring lens = 12 V, orifice 2 = 6 V. The ion guide was set to 200 V to detect ions of *m*/*z* 200 and higher.

### XPS Analysis

In order to further characterize the blue pigment distribution and provide information on the chemical state, a sample of blue paper was analyzed by X-ray photoelectron spectroscopy using a Thermo Scientific Nexsa X-ray photoelectron spectrometer (XPS) with a hemispherical analyzer and a monochromatic Al *K*_α_ source (1486.7 eV). First, the XPS survey spectra were collected using a pass energy of 150 eV, an energy step size of 1.0 eV, and a 20 ms/step dwell time. Then, the high-resolution spectra of Cu 2p, C 1s, O 1s, N 1s, Mg 1s, Al 2p, Si 2p, and Ca 2p core lines were collected using 50 eV pass-energy, 0.1 eV energy step size, and 100 ms/step dwell time. The base pressure of the analysis chamber during the data acquisition was <2.0 × 10^–7^ mBar. To further understand the distribution of Cu on the sample, a line scan was performed with high-resolution Cu 2p spectra recorded on ten analysis points over a distance of 37.7 mm. Recorded spectra were analyzed using ThermoAvantage v5.9922 software.

### Calculations and Statistical Analysis

Descriptive statistics were calculated for each element in the study samples using Microsoft Excel. Concentrations below the reporting limit, indicated by < values in Table 2, were assigned a numerical value of half the reporting limit for the purposes of these calculations. Distributions of elements in rolling papers were tested for consistency with the normal and log-normal distributions using the Komolgorov–Smirnoff (K–S) test with open-source software (AAT Bioquest). Elements, where more than 20% of the measured values were below the reporting limits (Ag, Be, Cd, Hg, Mo, Se, Sb, Th, and Tl), were excluded from this analysis.

Exposure potential was calculated based on the elemental concentration of each sample using daily paper consumption via smoking (i.e., daily use) as follows:
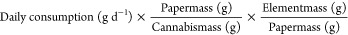


Daily use was estimated at both 2 and 5 g per day for a heavy and very heavy smoker, respectively. We consider estimates based on 5 g per day consumption to be conservative, as consumption rates for a daily smoker are likely to average ∼1 to 2 g d^–1^.^43^ While 5 g d^–1^ is excessive for most cannabis users, some users do self-report this level of cannabis use, and some state regulations are based on this level of consumption.^7^ Standard capacities of rolling papers were estimated based on manufacturers’ guidance as follows: 1 1/4 size, 0.75 g; King Slim, 1.05 g; King Size, 1.5 g. Using these estimates, a typical cannabis smoking product might be expected to contain ∼10 to 15% paper by mass, although this may vary based on the type of product and consumer preference. Additionally, our study did not directly measure exposure under realistic smoking conditions, so we did not correct our estimates for the fraction of uncombusted material (ash residual), etc. The combustion properties of the exterior rolling papers are not well-known, are likely dependent on the “paper” materials, and could further be dependent on the thickness of the paper and any additives. Therefore, our calculated exposure potentials can be considered a maximum exposure model similar to Zumbado et al.^18^ and have similar limitations.

## Supporting Information Available

Elemental composition of rolling papers analyzed in this study; SEM-EDS analysis of selected rolling papers; and DART-TOF spectra (PDF)
