# Supplementary material for: Elemental Composition of Commercially Available Cannabis Rolling Papers
Source: ACS Omega. 2024 Apr 20;9(17):19020–30. doi: 10.1021/acsomega.3c09580 (PMC11064008; doi:10.1021/acsomega.3c09580)
Supplement: Supplementary file 1 — ao3c09580_si_001.pdf [file ao3c09580_si_001.pdf]

The Elemental Composition of Commercially Available Cannabis Rolling Papers

Derek Wright[a *], Michelle M. Jarvie[a], Benjamin Southwell[a], Carmen Kincaid[a], Judy Westrick[b], S. Sameera Perera[b], David Edwards[c], and Robert B. Cody[c]

[a]School of Chemistry, Environmental, and Geosciences, Lake Superior State University, 650 W. Easterday Ave., Sault Ste. Marie, MI, 49783, USA

[b]Lumigen Instrument Center, Wayne State University, A. Paul Schaap Chemistry Building, 5101 Cass Ave, Detroit, MI 48202

[c]JEOL USA, 11 Dearborn Road, Peabody, MA 01960

*Corresponding author at: School of Chemistry, Environmental, and Geosciences, Lake Superior State University, 650 W. Easterday Ave., Sault Ste. Marie, MI, 49783, USA. E-mail address: dwright1@lssu.edu

Supporting Information: Additional experimental data including photographs of experimental setup

Table S1: Elemental composition of rolling papers analyzed in this study

	mg g^{-1}						µg g^{-1}																			
**Papers**	**Na**	**K**	**Ca**	**Mg**	**Fe**	**Al**	**Be**	**V**	**Cr**	**Mn**	**Co**	**Ni**	**Cu**	**Zn**	**As**	**Se**	**Mo**	**Ag**	**Cd**	**Sb**	**Ba**	**Hg**	**Tl**	**Pb**	**Th**	**U**
blend of natural fibers	0.15	0.26	2.5	0.33	0.01	0.05	<0.01	0.05	0.09	27.2	0.01	0.1	5.5	9.9	0.01	<0.2	<0.1	0.01	0.05	<0.01	9.8	<0.002	0.3	0.30	0.01	0.04
clear cellulose	1.03	0.06	0.1	0.03	0.01	<0.01	<0.01	0.01	0.08	0.2	0.01	<0.1	0.3	<0.1	0.01	<0.2	<0.1	<0.01	<0.01	<0.01	0.3	<0.002	<0.2	0.06	<0.01	<0.01
clear cellulose	1.70	0.02	0.2	0.02	0.01	<0.01	<0.01	0.02	0.14	0.1	0.01	<0.1	0.1	0.9	0.01	<0.2	<0.1	<0.01	<0.01	0.01	0.6	<0.002	<0.2	0.16	<0.01	0.01
grain fibers	1.03	0.04	68.6	0.52	0.10	0.10	0.02	0.66	1.50	11.5	0.04	0.3	28.7	1.2	0.22	<0.2	<0.1	<0.01	0.06	0.04	44.2	0.002	<0.2	0.10	0.02	0.10
asiatic cotton mallow	1.66	0.01	0.1	0.01	<0.01	<0.01	<0.01	<0.01	0.10	0.1	0.01	<0.1	0.1	<0.1	0.01	<0.2	<0.1	<0.01	<0.01	<0.01	0.8	<0.002	<0.2	0.08	<0.01	0.05
bamboo	0.12	0.28	2.7	0.96	0.04	0.13	<0.01	0.08	0.42	58.1	0.02	<0.1	3.2	4.0	0.01	<0.2	<0.1	<0.01	0.14	<0.01	12.2	<0.002	<0.2	0.17	0.01	0.02
flax	0.17	0.20	62.4	0.71	0.08	0.07	<0.01	0.11	1.20	15.6	0.02	0.3	3.0	1.2	0.04	<0.2	<0.1	0.01	0.02	0.01	11.0	<0.002	<0.2	0.15	0.01	0.04
flax	0.18	0.18	48.7	0.62	0.06	0.08	<0.01	0.13	0.64	17.3	0.02	0.3	2.9	1.4	0.04	<0.2	<0.1	<0.01	0.03	0.01	12.0	<0.002	<0.2	0.22	0.02	0.03
hemp	0.14	0.22	6.2	0.21	0.03	0.05	<0.01	0.03	0.46	6.4	0.01	0.1	2.7	1.0	0.02	<0.2	<0.1	<0.01	0.01	<0.01	8.2	0.002	<0.2	0.07	0.01	0.01
hemp	0.53	0.47	2.8	0.36	0.04	0.02	<0.01	0.20	3.02	0.8	0.03	1.0	3.3	0.5	0.03	<0.2	<0.1	0.01	<0.01	0.01	3.0	<0.002	<0.2	0.15	0.01	0.01
hemp	0.14	0.23	7.9	0.26	0.04	0.02	<0.01	0.04	0.48	8.7	0.01	0.1	3.1	1.3	0.04	<0.2	<0.1	<0.01	0.01	<0.01	10.3	<0.002	<0.2	0.11	0.01	0.01
hemp	0.16	0.45	1.5	0.23	0.02	0.01	<0.01	0.05	0.91	1.3	0.01	0.3	0.7	0.4	0.01	<0.2	<0.1	<0.01	<0.01	<0.01	2.7	<0.002	<0.2	0.04	<0.01	<0.01
hemp	0.16	0.49	1.5	0.22	0.02	0.01	<0.01	0.04	0.55	1.3	0.01	0.3	0.7	0.4	0.01	<0.2	<0.1	<0.01	<0.01	0.01	2.7	<0.002	<0.2	0.04	<0.01	<0.01
hemp, flavored, blue	0.58	0.24	44.1	0.39	0.04	0.92	<0.01	0.20	1.23	11.8	0.02	0.5	160.4	42.3	0.24	<0.2	0.4	0.01	0.01	0.19	3.9	<0.002	<0.2	0.40	0.01	0.06
hemp, flavored, green	0.87	0.21	43.9	0.35	0.04	0.28	<0.01	0.20	1.26	14.0	0.02	0.4	31.0	12.8	0.09	<0.2	0.1	<0.01	0.01	0.01	3.4	<0.002	<0.2	0.17	0.01	0.03
hemp, flavored, blue	1.18	0.29	33.6	0.32	0.11	0.29	<0.01	0.21	0.99	7.4	0.04	0.4	46.3	25.0	0.23	<0.2	0.2	0.02	0.03	0.02	2.7	<0.002	<0.2	0.27	0.02	0.07
hemp, flavored, purple	1.28	0.19	36.2	0.40	0.07	0.05	<0.01	0.15	3.12	18.6	0.03	1.4	168.3	1.9	0.04	<0.2	0.8	<0.01	0.03	0.01	6.0	<0.002	<0.2	0.14	0.01	0.03
hemp	0.13	0.23	5.6	0.19	0.04	0.05	<0.01	0.04	0.65	6.6	0.01	0.2	2.9	1.0	0.02	<0.2	<0.1	<0.01	0.01	0.01	9.0	0.003	<0.2	0.08	<0.01	0.01
hemp	0.19	0.41	1.7	0.29	0.01	<0.01	<0.01	0.03	0.58	0.7	0.01	<0.1	0.3	<0.1	0.01	<0.2	<0.1	<0.01	<0.01	<0.01	3.5	<0.002	<0.2	0.02	<0.01	0.01
rice	0.27	0.67	5.6	0.27	0.02	0.01	<0.01	0.05	1.52	2.3	0.02	<0.1	0.5	<0.1	0.01	<0.2	<0.1	<0.01	<0.01	<0.01	2.6	<0.002	<0.2	0.04	<0.01	0.01
rice	0.27	0.05	17.7	0.56	0.10	0.04	<0.01	0.31	6.56	10.4	0.09	1.4	2.5	<0.1	0.04	<0.2	<0.1	<0.01	0.02	0.01	2.1	<0.002	<0.2	0.13	<0.01	0.09
unspecified	0.73	0.62	48.9	0.66	0.10	0.15	<0.01	2.77	2.49	6.7	0.05	1.0	1.6	0.7	0.11	<0.2	<0.1	<0.01	0.01	0.01	4.9	<0.002	<0.2	0.12	0.03	0.14
unspecified	0.17	0.36	34.1	0.60	0.08	0.05	<0.01	0.19	3.53	20.3	0.05	1.2	1.9	1.7	0.05	<0.2	0.1	<0.01	0.03	0.01	5.4	<0.002	<0.2	0.14	0.02	0.04
unspecified	0.36	0.88	2.7	0.52	0.06	0.02	<0.01	0.06	3.66	14.9	0.04	0.4	0.9	1.0	0.02	<0.2	<0.1	<0.01	<0.01	0.01	5.9	<0.002	<0.2	0.05	<0.01	0.01
unspecified	0.02	0.11	0.4	0.05	0.01	<0.01	<0.01	0.02	0.19	2.8	<0.01	0.1	0.2	0.8	<0.01	<0.2	<0.1	<0.01	<0.01	<0.01	0.5	<0.002	<0.2	0.01	<0.01	0.01
unspecified	0.21	0.51	1.5	0.32	0.04	0.01	<0.01	0.03	2.30	6.8	0.02	0.3	0.6	0.5	0.01	<0.2	<0.1	<0.01	<0.01	<0.01	3.3	<0.002	<0.2	0.04	<0.01	0.01
unspecified	0.35	0.01	0.5	0.13	0.01	<0.01	<0.01	0.02	0.55	0.7	0.01	0.1	0.1	0.4	0.01	<0.2	<0.1	<0.01	<0.01	0.01	1.6	<0.002	<0.2	0.08	<0.01	<0.01
unspecified "plant-based"	0.12	0.28	2.9	0.19	0.07	0.02	<0.01	0.08	1.25	17.7	0.03	0.6	0.7	4.8	0.02	<0.2	<0.1	0.02	0.04	<0.01	3.3	0.002	<0.2	0.06	<0.01	0.02
wood pulp	0.35	0.48	1.5	0.33	0.01	0.01	<0.01	<0.01	0.59	0.8	<0.01	<0.1	0.3	<0.1	<0.01	<0.2	<0.1	<0.01	<0.01	<0.01	3.4	<0.002	<0.2	0.04	<0.01	0.01
**Filter Tip**																										
unspecified, white	0.79	0.02	114.0	2.43	0.19	0.38	<0.01	0.16	0.19	17.7	0.03	<0.1	10.1	1.4	0.10	<0.2	<0.1	<0.01	0.01	<0.01	2.3	<0.002	<0.2	0.35	0.02	0.07
**Cones**																										
clear cellulose, purple tip	0.85	0.02	53.6	0.57	0.06	0.19	<0.01	0.14	0.85	9.6	3.09	0.4	98.7	<0.1	0.03	<0.2	0.3	0.01	0.01	<0.01	1.7	<0.002	<0.2	0.13	0.03	0.10
wood fiber	0.31	0.10	1.0	0.64	0.72	1.84	0.01	3.28	3.86	14.4	0.30	<0.1	2.1	6.1	0.09	<0.2	0.1	0.01	0.03	0.01	8.0	<0.002	<0.2	0.23	0.09	0.19
bamboo	0.19	0.14	0.8	0.56	0.52	1.90	<0.01	5.31	7.25	19.8	0.17	0.6	2.8	5.2	0.09	<0.2	0.3	0.01	0.03	0.01	4.4	0.003	<0.2	0.24	0.17	0.16
hemp	0.41	0.04	47.5	0.43	0.17	0.52	<0.01	0.27	0.58	12.9	0.09	0.3	1.0	1.8	0.09	<0.2	<0.1	0.01	0.04	<0.01	11.2	0.004	<0.2	0.44	0.06	0.09
hemp blend, 24k edible gold	0.22	0.09	22.9	0.34	0.09	0.71	0.01	0.35	1.17	3.4	0.04	0.5	138.7	13.4	0.12	<0.2	0.1	161.47	0.01	0.04	2.2	0.015	<0.2	0.42	0.13	0.05
unspecified	0.41	0.05	92.0	1.59	0.12	0.57	0.04	0.38	1.37	21.8	0.11	0.3	6.1	1.3	0.22	<0.2	4.3	0.06	0.04	0.01	15.0	<0.002	<0.2	0.33	0.09	0.09
unspecified	0.29	0.03	116.4	0.96	0.16	0.26	<0.01	0.37	0.34	27.5	0.04	<0.1	1.0	<0.1	0.06	<0.2	<0.1	<0.01	<0.01	0.01	14.0	0.005	<0.2	0.35	0.06	0.05
unspecified "plant-based"	0.30	0.05	36.2	0.25	0.11	0.18	0.01	0.36	0.54	38.3	0.20	0.7	11.9	2.4	0.05	<0.2	<0.1	<0.01	0.03	0.01	21.3	<0.002	<0.2	0.17	0.03	0.01
goji berry	0.76	1.63	2.2	0.23	0.06	0.05	<0.01	0.05	2.49	1.9	0.03	0.5	1.9	20.9	0.06	<0.2	0.2	0.01	0.02	<0.01	2.6	<0.002	<0.2	0.06	0.02	0.01
hemp, red	0.16	0.06	62.8	1.24	0.09	0.72	<0.01	0.57	1.63	17.0	0.03	0.5	0.7	3.8	0.09	<0.2	<0.1	<0.01	0.07	11.19	91.7	0.043	<0.2	0.26	0.05	0.17
hemp, black	1.23	0.05	25.7	0.74	0.29	2.86	0.04	1.64	1.20	18.1	0.20	1.2	43.0	11.1	0.20	<0.2	0.5	0.01	0.05	0.05	55.0	0.023	<0.2	1.20	0.07	0.06
hemp, yellow	0.17	0.06	64.9	1.23	0.09	0.75	0.01	0.55	1.61	17.3	0.03	0.5	0.8	3.4	0.08	<0.2	<0.1	0.01	0.08	10.43	1.5	0.043	0.4	0.30	0.05	0.17
hemp, blue	0.18	0.06	62.2	1.23	0.09	0.94	<0.01	0.58	2.48	16.8	0.03	0.5	250.9	6.1	0.16	<0.2	0.2	0.02	0.09	7.60	1.4	0.045	<0.2	0.46	0.09	0.18
hemp, green	0.17	0.06	63.9	1.20	0.09	0.78	0.01	0.55	1.71	17.3	0.03	0.5	50.6	4.9	0.11	<0.2	1.4	0.02	0.08	11.45	1.7	0.05	<0.2	0.31	0.04	0.16
hemp, purple	0.17	0.05	66.1	1.26	0.10	0.76	<0.01	0.58	1.79	17.7	0.03	0.6	48.9	3.6	0.12	<0.2	33.2	0.01	0.09	10.92	5.1	0.05	<0.2	0.28	0.05	0.17
palm pulp, blue	0.99	0.31	61.1	13.54	0.09	1.39	<0.01	0.33	1.87	13.1	0.16	0.5	198.0	8.2	0.09	<0.2	0.1	0.01	0.05	11.72	148.6	0.144	<0.2	0.32	0.03	0.21
palm pulp, rainbow	1.07	0.23	54.7	12.66	0.09	1.51	<0.01	0.26	6.34	16.5	0.05	0.6	243.2	3.2	0.07	<0.2	0.1	0.01	0.05	10.00	50.8	0.172	<0.2	0.27	0.04	0.21
unspecified, light blue	0.33	0.04	80.2	1.34	0.09	0.39	0.03	0.30	0.92	19.1	0.11	0.2	10.4	3.2	0.17	<0.2	3.6	0.01	0.04	0.01	1.8	<0.002	<0.2	0.28	0.06	0.07
**Wraps**																										
Asiatic Cotton Mallow	6.29	0.02	0.1	0.01	0.02	<0.01	<0.01	0.01	0.41	0.2	0.35	0.2	0.1	<0.1	0.01	<0.2	<0.1	<0.01	<0.01	<0.01	0.2	<0.002	<0.2	0.14	<0.01	<0.01
Hemp	2.63	14.82	37.9	7.04	0.28	0.15	<0.01	0.34	1.24	82.4	0.13	2.1	9.8	38.4	0.11	<0.2	1.5	0.01	0.13	0.02	23.9	0.009	<0.2	0.53	0.04	0.02
Hemp	6.49	3.49	46.3	0.81	0.14	0.11	0.02	0.54	8.47	17.7	0.33	4.9	3.7	6.7	0.19	<0.2	0.1	<0.01	0.04	0.02	3.4	0.01	<0.2	0.20	0.03	0.20
Hemp	2.72	9.21	27.5	4.62	0.18	0.07	<0.01	0.21	0.66	43.1	0.07	0.6	9.0	26.5	0.04	<0.2	1.2	<0.01	0.02	0.02	10.9	0.05	<0.2	0.57	0.02	0.01
palm leaf	0.06	16.21	13.4	4.97	0.04	0.02	<0.01	0.03	0.00	137.7	0.21	0.4	6.8	10.4	0.02	<0.2	<0.1	<0.01	0.01	0.03	10.1	0.019	<0.2	0.17	<0.01	<0.01
**Minimum Reportable Value**	0.002	0.01	0.050	0.001	0.01	0.01	0.01	0.01	0.05	0.1	0.01	0.1	0.02	0.1	0.01	0.2	0.1	0.01	0.01	0.01	0.003	0.002	0.2	0.02	0.01	0.01

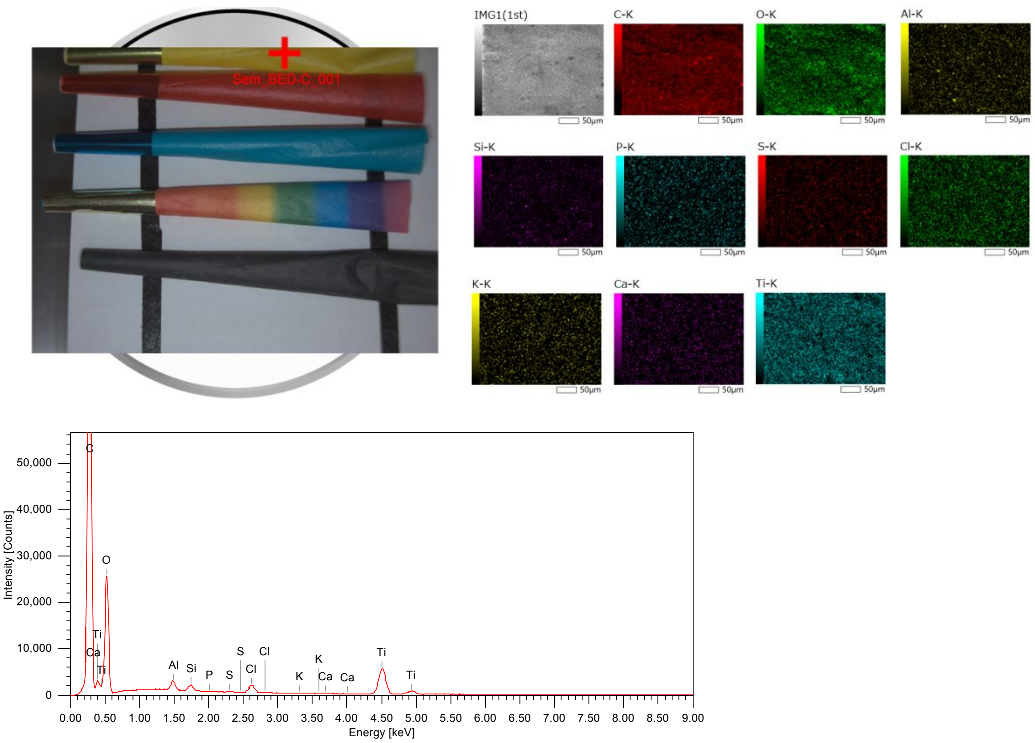

Figure S1. SEM-EDS Analysis of the yellow cone

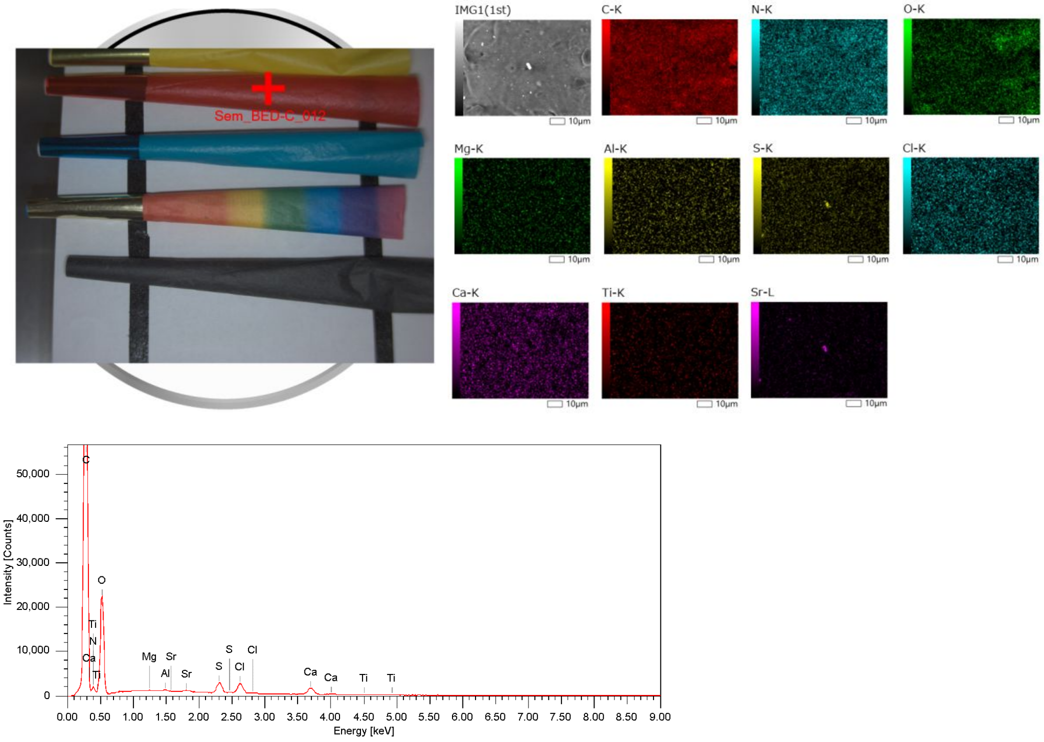

Figure S2. SEM-EDS Analysis of the red cone

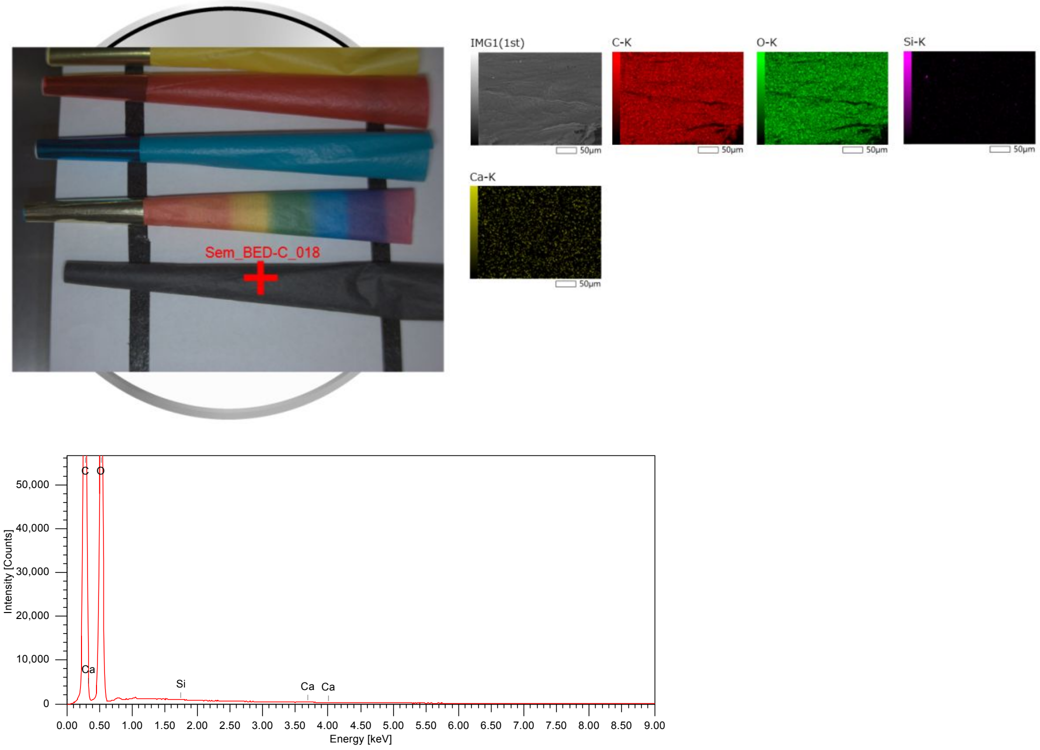

Figure S3. SEM-EDS Analysis of the black cone

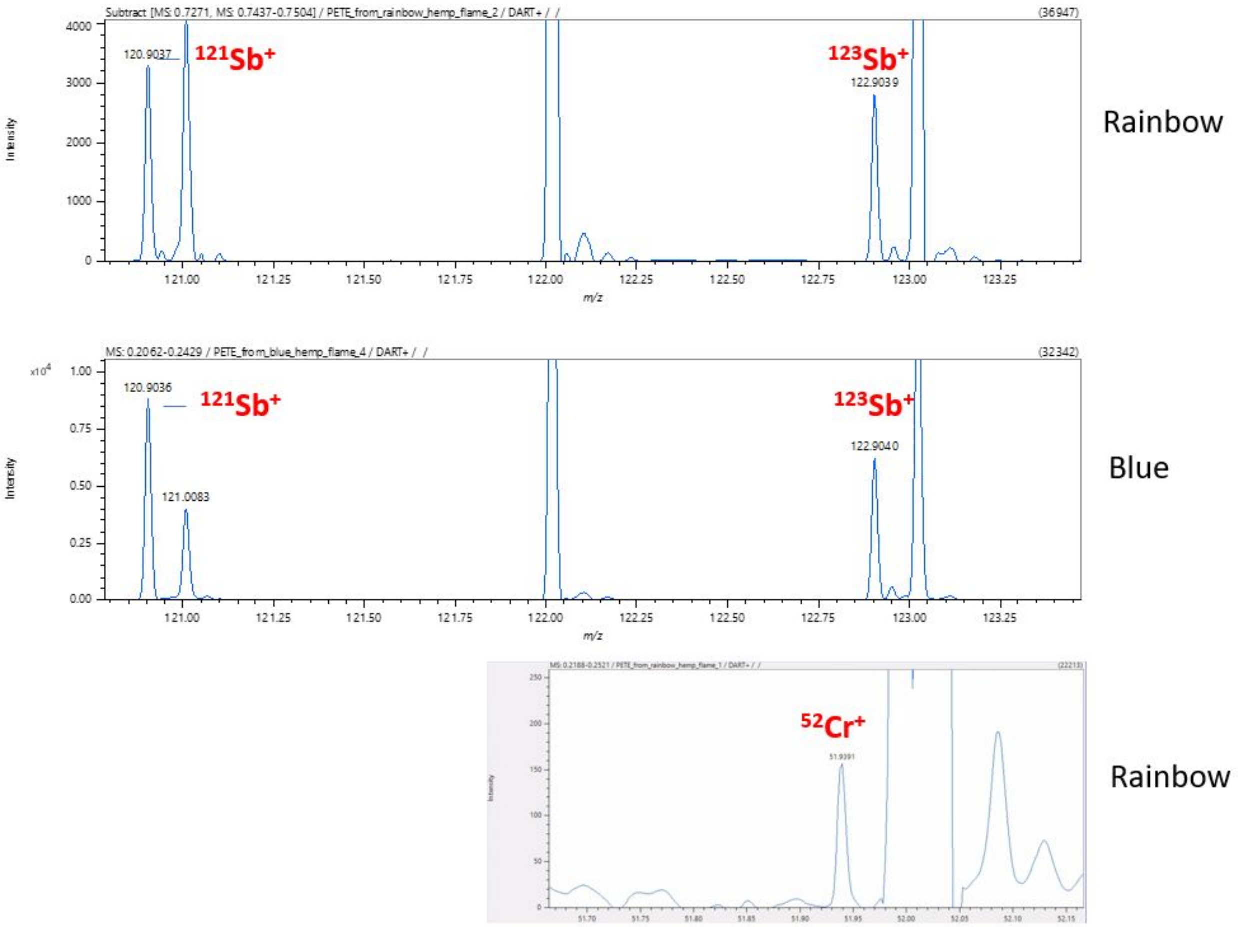

Figure S4. AccuTOF-DART flame ionization showing Antimony and Chromium in PETE base
